# Three-dimensional nuclear telomere architecture and differential expression of aurora kinase genes in chronic myeloid leukemia to measure cell transformation

**DOI:** 10.1186/s12885-022-10094-5

**Published:** 2022-09-29

**Authors:** Fábio Morato de Oliveira, Valderez Ravaglio Jamur, Lismeri Wuicik Merfort, Aline Rangel Pozzo, Sabine Mai

**Affiliations:** 1 Laboratory of Medical Genetics, Câmpus Jatobá - Cidade Universitária, Federal University of Jataí, BR 364, km 195, n° 3800, Jataí, CEP 75801-615 Brazil; 2 Complexo Hospital das Clínicas, Universidade Federal do Paraná, Curitiba, Paraná 80060-240 Brazil; 3 Research Institute in Oncology and Hematology CancerCare Manitoba, The Genomic Centre for Cancer Research and Diagnosis, The University of Manitoba, Winnipeg, MB R3E 0V9 Canada

**Keywords:** CML, Telomere, Nuclear architecture, Genomic instability

## Abstract

**Background:**

Telomere dysfunction results in aneuploidy, and ongoing chromosomal abnormalities. The three-dimensional (3D) nuclear organization of telomeres allows for a distinction between normal and tumor cells. On the other hand, aurora kinase genes (*AURKA* and *AURKB*) play an important role regulating the cell cycle. A correlation between overexpression of aurora kinase genes and clinical aggressiveness has been demonstrated in different types of neoplasias. To better understand cellular and molecular mechanisms of CML evolution, it was examined telomere dysfunction (alterations in the 3D nuclear telomere architecture), and the expression levels of *AURKA* and *AURKB* genes in two clinical distinct subgroups of CML samples, from the same patient.

**Methods:**

Eighteen CML patients, in total, 36 bone marrow samples (18 patients, *chronic* vs. *accelerated*/*blast phase*) were eligible for 3D telomeric investigations. Quantitative 3D imaging, cytologic diagnosis and cytogenetic determination of additional chromosomal abnormalities were assessed according to standard protocols.

**Results:**

Using TeloView software, two CML subgroups were defined based on their 3D telomeric profiles, reflecting the different stages of the disease (*chronic* vs. *accelerated*/*blast phase*). Statistical analyses showed significant differences between the CML subgroups (*p* < 0.001). We also found that *AURKA* and *AURKB* mRNA were expressed at significantly higher levels in both CML subgroups, when compared with healthy donors. Our findings suggest that the evolution of CML progresses from a low to a high level of telomere dysfunction, that is, from an early stage to a more aggressive stage, followed by disease transformation, as demonstrated by telomere, additional chromosomal abnormalities, and gene expression profile dynamics.

**Conclusions:**

Thus, we demonstrated that 3D telomere organization, in accordance with the genomic instability observed in CML samples were able to distinguish subgroup CML patients. Classifying CML patients based on these characteristics might represent an important strategy to define better therapeutic strategies.

## Background

Telomeres are specialized structures presents at the end of each chromosome, composed of long sequences of TTAGGG repeats, able to maintain integrity and stability of the genome [1, 2]. Telomeres also act protecting the cell from progressive DNA shortening during cell division, and they are essential to prevent improper fusion of chromosomes. On the other hand, dysfunctional telomeres indistinguishable from damaged DNA contribute to the ultimate protection of chromosomes lead to genome instability through fusion-fusion bridge cycles increasing genomic instability, ultimately leading to cancer development [3, 4].

Each cell division promotes the loss of telomere repeats due the “end-replication” problem. To partially solve this situation, the telomere length is maintained by an enzyme called telomerase, which is overexpressed in germ cells and neoplastic tissue [1, 5]. Some investigations have pointed that cell with shortest telomeres are preferably chosen by telomerase for elongation process [1, 5, 6]. On the other hand, during replicative senescence, in the absence of telomerase activity, the telomeres eventually shorten, and the cell begins cell cycle arrest, at G1 phase [6]. In this scenario, the telomere size reduction limits the dynamics of somatic cell division and may provide a tumor suppressor mechanism. However, when cells get critically short telomeres, replicative senescence precludes them from end-to-end fusions, that could eventually result in aneuploidy, and tumorigenesis [6, 7].

The telomere architecture, as a reflection of telomere shortening and telomerase activation, has been considered an important biomarker for cell transformation [8, 9]. The 3D nuclear architecture of telomeres has been shown to be able to distinguish between normal and malignant cells. The 3D remodeling of the nuclear space has been also associated with genomic instability in cancer cells [8, 10, 11]. The key elements to evaluate telomere related genomic instability are based on the following steps (1) the number of telomeres (telomere signals), (2) telomere length (telomere signal intensity), (3) the number of telomere aggregates (TAs), (4) telomere distribution within a nucleus, and (5) telomere positions (the distance of each telomere from the nuclear center versus the periphery) [9, 12, 13].

Chronic myeloid leukemia (CML) represents a common form of leukemia, characterized by a proliferation of myeloid cell lineage. From the genetic point of view, this condition presents a chromosome translocation, involving the chromosomes 9 and 22, originating the so-called Philadelphia (Ph) chromosome [t(9;22) (q34;q11.2)], which generates a chimerical gene *BCR-ABL* [14]. The presence of the Ph chromosome in CML, at diagnosis and the presence of additional chromosomal abnormalities along the course of the disease constitutes important markers for evaluation of the disease progression [14, 15]. On the other hand, molecular mechanisms underlying the CML evolution are not completely understood. Some studies have demonstrated an increased activity of the fusion protein BCR::ABL1, followed by inactivation of tumor suppressor genes, as for example *TP53*, a default in DNA repair machinery, an emergence of other chromosomal abnormalities, and occurrence of genomic instability. However, their role in the evolution of CML remains unknown [16]. In CML, the telomeres are significantly shorter when compared to telomeres from normal cells [16, 17]. Is it also evident the cells from patients in accelerated/blast phase show significantly telomere shortening when compared with CML in chronic phase or in cytogenetic remission [18, 19]. This information allows us to suggest, that the dynamics of telomere architecture in CML could be associated with disease progression, and it may represent an important biomarker for disease progression.

Aurora kinases are a family of serine/threonine kinases that plays an important role in normal mitosis by executing various cells divisions and maintaining the integrity of the genome [20, 21]. The Aurora family consists of three members, *AURKA*, *AURKB* and *AURKC*, which share 67–76% amino acid sequence identity in their catalytic domains and few similarities in their N-terminal [20–22]. In various malignancies, including colon and breast cancer, the expression levels of *AURKA* and *AURKB* are generally high [23–26]. It has been also demonstrated that the levels of these genes are elevated in a variety type of hematological malignant cells, including CML [24]. Based on previously mentioned, it is possible to affirm that the differential expression of aurora kinase genes may offers a great contribution to be used as biomarkers of cell transformation [24, 25]. In conditions in which the clinical/morphological classification determines exclusive therapeutic ways, the comparison among the levels of *AURKA* and *AURKB*, in different stages of the disease may indicate cellular progression form a pre-neoplastic form to neoplastic status [24, 25].

In the present study, we show direct evidence that quantitative FISH analysis, 3D nuclear architecture of telomeres and the differential expression of aurora kinase genes were able to distinguish two distinct groups of CML patients, based on clinical/morphological classification (chronic vs. accelerated/blast phase).

## Methods

### Samples

Thirty-six bone marrow samples from CML patients were obtained after written informed consent before participating in the study (age ranged from 41 to 52). For each patient, we collected sample in chronic and accelerated/blast phase. The laboratorial diagnoses were confirmed by the presence of the Philadelphia (Ph) chromosome as the sole abnormality (*chronic phase*), and the presence of additional chromosomal abnormalities for accelerated/blast phase of CML. We used *G-banding* analysis and fluorescence in situ hybridization (FISH) in all samples, to confirm the presence of the *Philadelphia* chromosome. In addition, fourteen blood samples from health donors were used as control. Informed consent was obtained from all the patients in accordance with the declaration of Helsinki. This study received approval by the Research Ethics Board on human studies in Brazil (CAE: 87629318.3.0000.0096).

### Co-Imuno quantitative fluorescent in situ hybridization (Q-FISH)

The bone marrow smears slides were fixed with 3.7% formaldehyde/1 × PBS for 20 min, washed three times with 1 × PBS for 5 min, and blocked with 4% BSA in 4× saline-sodium citrate (SSC) for 15 min. The cells were incubated with rabbit anti-CD34 antibody (ab81289, ABCAM, Cambridge, United Kingdom) as a primary antibody and Goat Anti-Rabbit IgG H&L-Alexa Fluor® 488 (ab150077, ABCAM, Cambridge, United Kingdom) as a secondary antibody. For Q-FISH, briefly, slides were incubated in 3.7% formaldehyde/1xPBS solution for 10 minutes, and after the slides were soaked in 20% glycerol/ 1xPBS solution for 45 min. The cells were treated by four repeated cycles of freeze-thaw in glycerol. After, the slides were incubated in 0.1 HCL solution and fixation in 70% formamide/2xSCC for 1 hour. For hybridization, slides were covered with 8 μl of PNA telomeric probe (Agilent Dako, Santa Clara, California, USA), sealed with coverslip and rubber cement. For denaturation, the slides were placed on a hot plate, protected from direct light, for 3 minutes, at 82 °C. The hybridization was carrying out for 2 hours, at 30 °C. The slides were then washed three times in 70% formamide/10 mM Tris (pH 7.4) solution, for 15 minutes followed by washing in 1xPBS at room temperature for 2 minutes, while shaking and in 0.1xSSC at 55 °C for 5 minutes while shaking. Finally, the slides were washed in 2x SSC/ 0.05% Tween 20 solution for three times, for 5 minutes, at room temperature while shaking. After final all cycles of washing, the nuclei were counter-stained with 4′,6-diamino-2-phenylindole (DAPI) (0.1 μg/ml) and antifade reagent (ThermoFisher, USA), and covered with coverslip for image acquisition [27, 28].

### 3D image acquisition and analysis using TeloView™

For each sample were analyzed 30 CD34+ interphase nuclei, using an AxioImager M1 microscope (Carl Zeiss, Jena, Germany), coupled to an AxioCam HRm charge-coupled device (Carl Zeiss, Jena, Germany) and a 63-x oil objective lens (Carl Zeiss, Jena, Germany). The acquisition times was 500 milliseconds (ms) for Cy3 (telomeres) and 5 ms for DAPI (nuclei). Sixty z-stacks were acquired at a sampling distance of *x*,*y*: 102 nm and *z*: 200 nm for each slice of the stack. AxioVision 4.8 software (Carl Zeiss, Jena, Germany) was used for 3D image acquisition. Deconvolved images were converted into TIFF files and exported for 3D-analysis using the TeloView™ (3D Signatures Inc.) software [29].

### Aurora kinase mRNA analysis

RNA was isolated from CML samples using TRIzol reagent (ThermoFisher, USA) according previously described [30]. Complementary DNA (cDNA) was synthesized from ~ 1 μg of total RNA using a High-Capacity cDNA reverse transcription Kit (Applied BioSystems, Foster City, CA, USA), following the manufacturer's instructions. For analysis of aurora kinase genes, primers and probes were developed by Assay on Demand (*AURKA*: Hs00269212_m1 and *AURKB*: Hs00177782_m1; Applied BioSystems). The *AURKA* and *AURKB* genes and *GAPDH* mRNA, used as endogenous internal control for each sample, were analyzed in duplicate on the same MicroAmp optical 96-well plates using a 7500 Real-Time PCR System (Applied BioSystems).

Real-time quantitative polymerase chain reaction (RQ-PCR) assays were performed in a final reaction volume of 20 μl. The comparative cycle threshold (Ct) method was used to determine the relative expression level of *AURKA* and *AURKB* genes. On comparative analysis of CML samples and healthy donors, *AURKA* and *AURKB* gene expression was calculated as a relative quantification to the *GAPDH* housekeeping gene. The gene expression *AURKA* and *AURKB* from CML samples was calculated as relative quantification to normal controls (ΔΔCt = ΔCt _patient_ – ΔCt _healthy donors+_) and expressed as 2^−ΔΔCt^, according previously described [30]. All primers were standardized by conventional semi-quantitative PCR analysis before proceeding to the real-time quantitative analysis.

### Data image analysis – 3D telomere architecture

To compare telomere architecture between the different stages of CML we used the software TeloView (3D Signatures Inc.) [29]. TeloView® measures six distinct parameters for each sample: (1) the number of telomeres (signals); (2) the total intensity (telomere size); (3) the distribution and frequency of telomere aggregates, which means clusters of telomeres that are found in proximity that cannot be further resolved; (4) the a/c ratios represent a measure defined by the cell cycle progression through interphase cells [8]. Thus, it is possible to check if there was a difference in cell cycle between the CML samples groups. The telomere dynamics varies according to the stages of the cell cycle (G0/G1, S, and G2); (5) Finally, TeloView (3D Signatures Inc.) software allows us to measure the distance of each telomere signal from the nuclear center versus the periphery [29]. For both CML groups, a graphical representation was obtained showing the distribution of the intensity of the acquired telomere fluorescent signals, the distribution of the frequency of telomere aggregates per cell and the acquired signals per cell.

### Statistical analysis for telomere architecture

Two distinct subgroups were defined based on their 3D telomeric profiles (*chronic* vs. *blastic phase*). The telomeric parameters (number, length, telomere aggregates, nuclear volumes, and *a/c* ratio) were compared between these subgroups using analysis of variance. Distribution of all telomere parameters in both subgroups was compared using chi-square analysis. Cell parameters averages were analyzed between subgroups with nested factorial analysis of variance taking both patient and cellular variations into account. Significante level was set at 0.05.

## Results

The purpose of this investigation was to perform a pilot study with 36 bone marrow CML samples from 18 patients (*chronic* vs. *blast phase*), to define the 3D nuclear telomeric profiles based on telomere numbers, telomeric aggregates, telomere signal intensities, nuclear volumes, and nuclear telomere distribution for each phase. In addition, we also obtained the expression profile for *AURKA* and *AURKB* genes in eight patients (*chronic* vs. *blast phase*). For both analysis (telomere and gene expression) we blindly subdivided the CML patients into 2 groups. The cytogenetic profile of 18 CML patients are shown in Table 1.

**Table 1:** Cytogenetic profile of CML patients during the two distinct phases of the disease (chronic vs. accelerated/blast phase)

CML samples	Chronic phase	Accelerated/Blast phase
CML 01	46,XY,t(9;22)(q34;q11.2)[20]	46–47,XY,+ 8[5],t(9;22)(q34;q11.2)[20]
CML 02	46,XX,t(9;22)(q34;q11.2)[20]	46–48,XX,+ 8[7],+ 19[2],t(9;22)(q34;q11.2)[20]
CML 03	46,XX,t(9;22)(q34;q11.2)[20]	46–47,XX,+ 8[12],t(9;22)(q34;q11.2)[16]
CML 04	46,XX,t(9;22)(q34;q11.2)[20]	46,XX,t(9;22)(q34;q11.2)[14],del(13)(q14q32)[7]
CML 05	46,XY,t(9;22)(q34;q11.2)[20]	47,XY,+i(9)(q10),t(9;22)(q34;q11.2)(q34;q11.2)[16]
CML 06	46,XY,t(9;22)(q34;q11.2)[20]	44,XY,der(5;17)(q10;q10),-7,add(20)(q13)[16]
CML 07	46,XY,t(9;22)(q34;q11.2)[20]	47,XY,+ 8,t(9;11)(p21–22;q23)
CML 08	46,XY,t(9;22)(q34;q11.2)[20]	47,XY,del(6)(q23q25),+ 7[20]
CML 09	46,XY,t(9;22)(q34;q11.2)[28]	46,XY,del(7)(q22;qter),t(9;22)(q34;q11.2)[13]/46,XY[2]
CML 10	46,XX,t(9;22)(q34;q11.2)[22]	46,XX,t(9;22)(q34;q11.2),del(20)(p11)[17]/46,XX,del(20)(p11)[3]
CML 11	46,XY,t(9;22)(q34;q11.2)[22]	46,XY,t(9;22)(q34;q11.2)[5]/46,XY,t(11;22;X)(q13;q11.2;p22.3)[20]
CML 12	46,XX,t(9;22)(q34;q11.2)[20]/46,XX[10]	46,XX,t(9,22)(q34;q11.2)[1]/47,XX,+ 8[2]/46,XX[17]
CML 13	46,XY,t(9;22)(q34;q11.2)[28]	46,XY,t(9,22)(q34;q11.2)[5]/47,XY,+ 8[21]
CML 14	46,XY,t(9;22)(q34;q11.2)[22]	47,XY,+ 8,t(9;22)(q34;q11.2),iso(17q)[20]
CML 15	46,XX,t(9;22)(q34;q11.2)[30]	46,XX,t(9,22)(q34;q11.2)[4]/48,XX,+ 8,t(9;22)(q34;q12),+ 19[20]
CML 16	46,XX,t(9;22)(q34;q11.2)[21]	46,XX,t(9,22)(q34;q11.2)[5]/48,XX,+ 8,t(9;22)(q34;q11.2),iso(17q),+ 21[18]
CML 17	46,XY,t(9;22)(q34;q11.2)[23]	45,XY,-7,t(9;22)(q34;q11.2)[20]
CML 18	46,XY,t(9;22)(q34;q11.2)[27]	48,XY,+ 8,t(9;22)(q34;q12),+ 19[20]

**Fig. 1:**
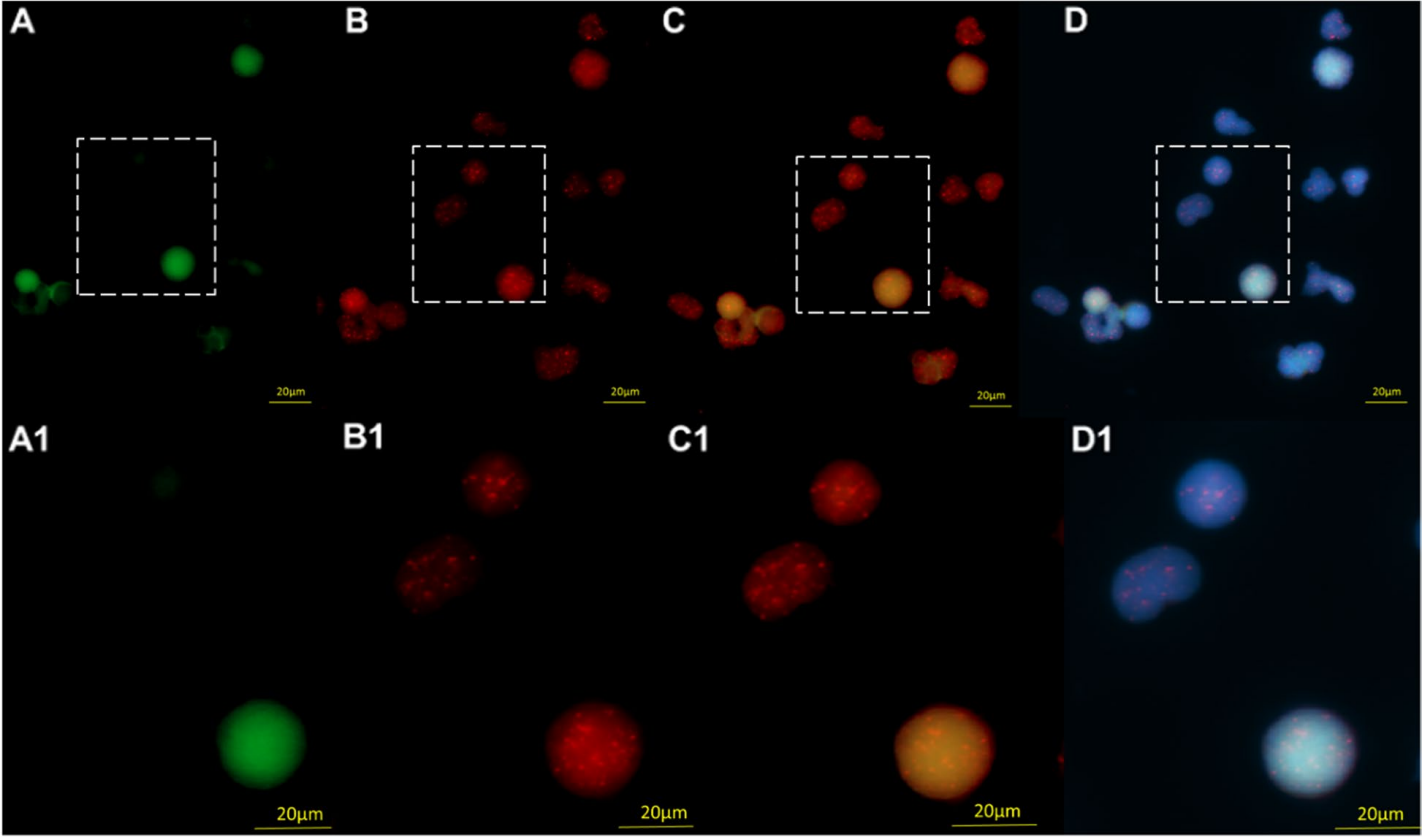
The CML CD34 positive cells fluoresce green, whereas the other hematopoietic cells remained unstained (**A**). The telomeres, hybridized with Cy3-labeled PNA probes, appear as red signals (**B**). **C** Merge of FITC (CD34) and Telomeres (CY3). The nuclei are counterstained with DAPI (blue) (**D**)

CML is a myeloproliferative neoplasm characterized by the expansion of the early hematopoietic progenitor cells pool which express CD34 antigen [31]. CML cells were differentiated from other hematopoietic cells based on the intensity of green fluorescence signals emitted by the CD34 antibody (Fig. 1).

**Fig. 2:**
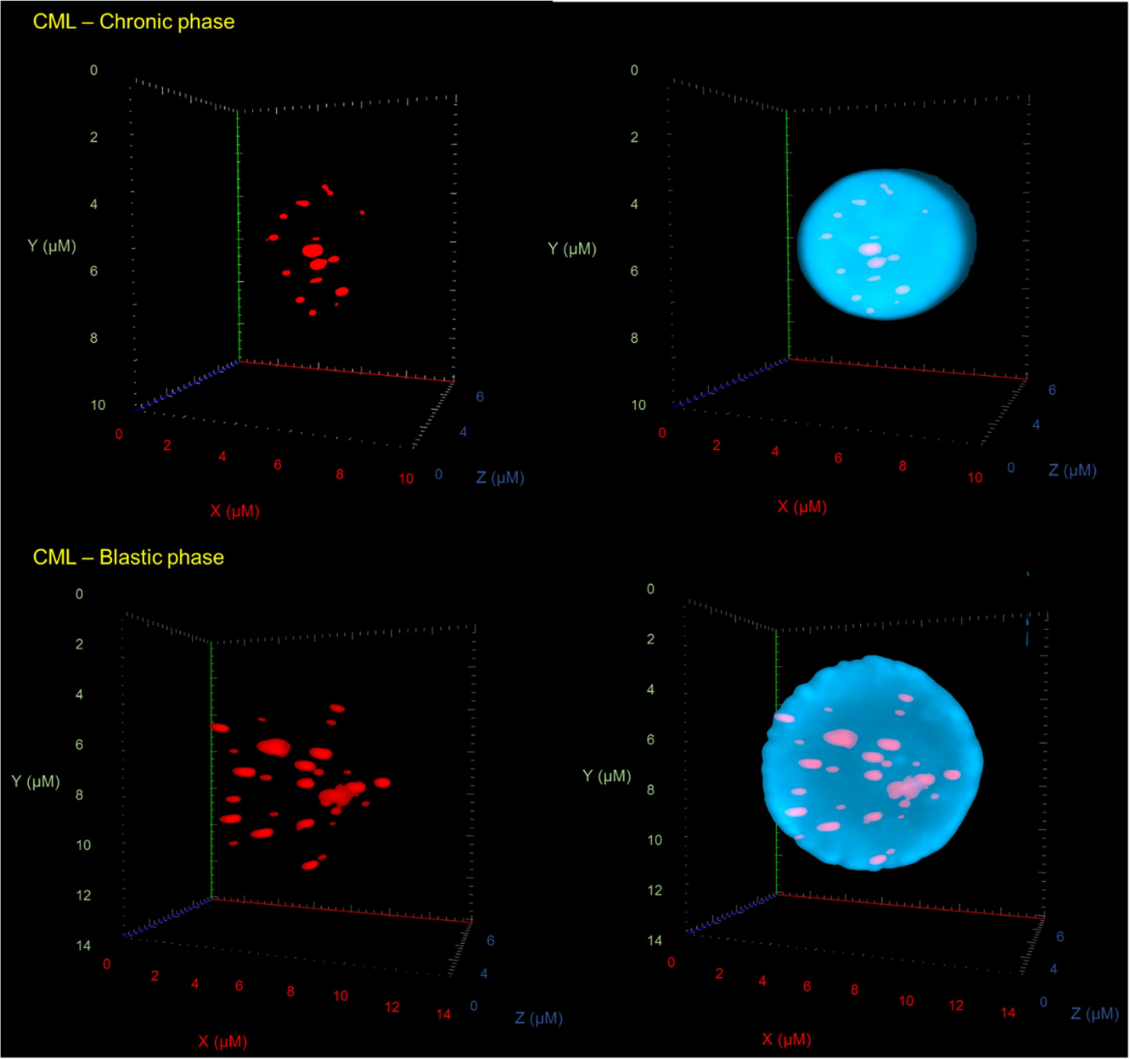
Evolution of 3D nuclear architecture in CML. The left side shows representative 3D nuclear telomere distribution (red) within the counterstained nucleus (blue). The number of telomere signals, the number of telomere aggregates and the nuclear volume increase

To examine the 3D nuclear telomere architecture in CML cells, 3D Q-FISH was performed. After image acquisition and deconvolution, 30 nuclei for each time point were analyzed with TeloView (3D Signatures Inc.) to determine the parameters reported in Material and Methods. The telomeres were visualized as red dots (Fig. 2) [29].

**Fig. 3:**
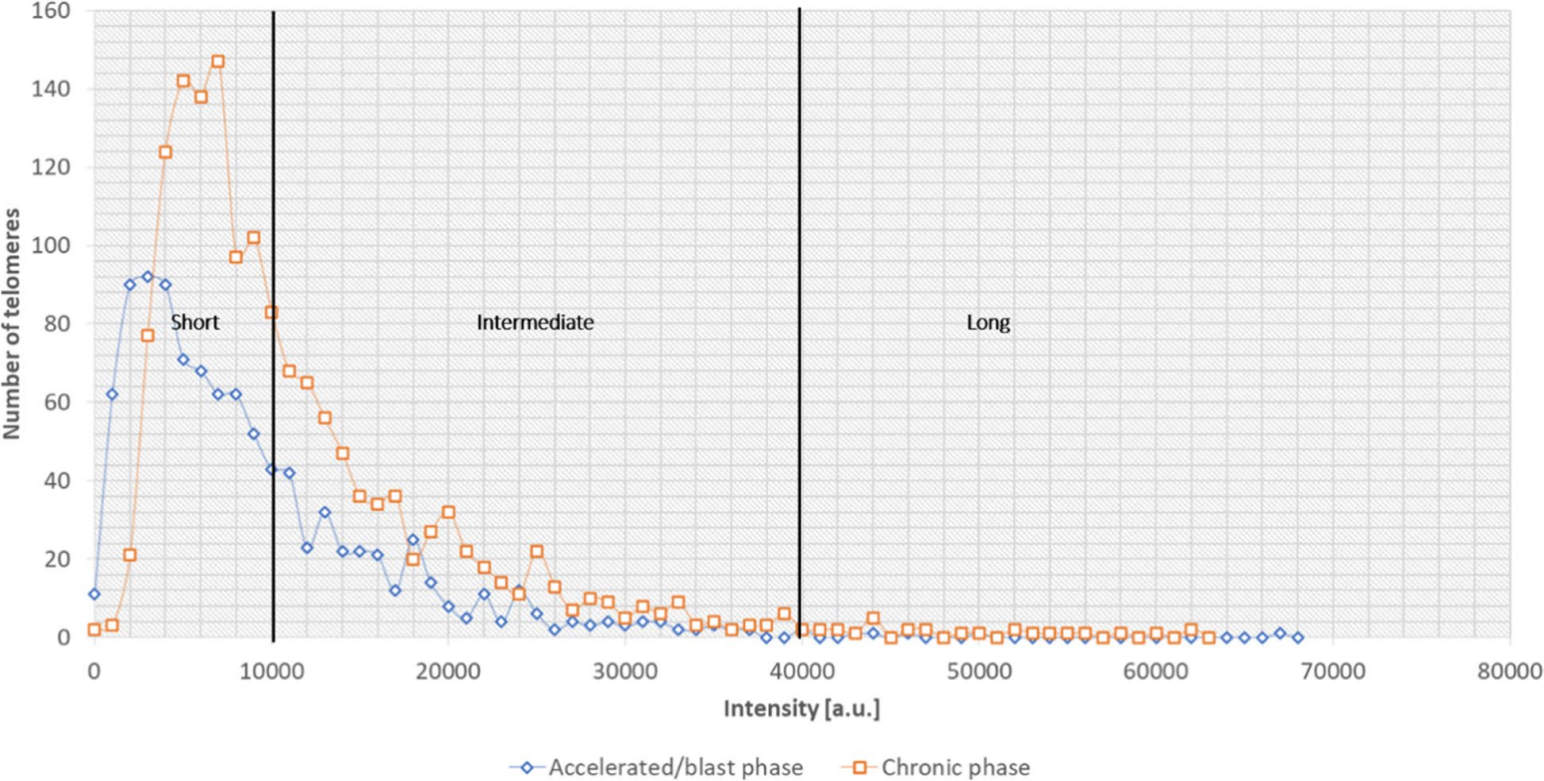
Graph distribution of number of telomeres according to their intensity (length of telomeres) for two samples from the same patient (chronic vs. accelerated/blastic phases). The image represents the 3D telomere distribution of the 3D telomeric profile. Black bars separate the 3 cell populations with short, intermediate, and long telomeres, respectively

For each CML samples we obtained the 3D telomere profiles, considering the total number of telomeres, intensity, lengths of each telomere and the number of telomeres aggregates per cell. For analysis of each sample, it was used the software TeloView (3D Signatures Inc.) [29]. The organization pattern of telomeres per sample made it possible to identify in each CML sample cell populations with short, intermediate, and long telomeres (Fig. 3). Based on this characteristic, it was able to stratify the same profiles in distinct subgroups. According to 3D telomere profiles, two CML subgroups were stablished based on blindly analysis and without any information about the status of the disease (*chronic* vs. *blastic phase*). We noted that all eighteen samples clinically classified as chronic phase exhibited a similar 3D telomere profile. On the other hand, a distinct profile was observed among the patient samples classified as blastic phase. Statistical analyses showed significant differences between the two CML subgroups (*p* < 0.001). Considering the telomere parameters investigated, all of them showed significant differences between CML chronic phase and CML blastic phase (Table 2). Considering that the samples analyzed, in the different stages of the disease, belong to the same patient, the telomere dynamic observed (intensities, number, and frequency of telomere aggregates) may represent a clonal evolution of the disease.

**Table 2:** Telomere parameters according to CML subgroups

CML patients	Total number of telomeres (mean ± SD)	Total number of telomere aggregates (mean ± SD)	Total intensity (mean ± SD)	Intensity of all signals (mean ± SD)	a/c ratio (mean ± SD)	Nuclear volume (mean ± SD)
Chronic phase	39,45 ± 6,49	3,37 ± 1,19	414,618,5 ± 235,693,0	13,889 ± 2254	2,55 ± 0,48	191,790 ± 85,724
Accelerated/ blast phase	41,51 ± 5,25	4,73 ± 0,92	512,852,4 ± 155,272,4	14,574 ± 2739	6,65 ± 1,97	328,714 ± 134,704
*p.value between chronic and accelerated CML phases*	*p* < 0,001	*p* < 0,001	*p* < 0,001	*p* < 0,001	*p* < 0,0001	*p* < 0,0001

From the cytogenetic point of view, two subgroups were stablished (Table 1), and we found patients with distinct chromosomal abnormalities during the blastic phase of the disease. All eighteen patients exhibited additional chromosomal abnormalities after comparison between chronic and blast phase, indicating cumulative acquisition. However, in our analysis we noted that only three patients did not show the presence of Ph chromosome in blastic phase (Table 1). The cytogenetic profile of the samples was important to discriminate the patient subgroups status and was consonant with the 3D nuclear telomeric obtained. The number of signals per nucleus, telomere aggregates per nucleus, total telomere intensity, and average intensity of signals were all higher in blastic phase, as compared with chronic phase (*p* < 0.0001). Thus, in addition to cytogenetic profile we observed that telomere parameters represent a fully accurate tool to distinguish both CML groups.

In the context of telomere dynamics, it is also possible to obtain information regarding cell cycle progression, considering nuclear volume, distribution of the telomeres per nuclear volume and the a/c ratio. In our results, the nuclear volume showed itself significantly higher in blastic phase than chronic (*p* < 0.0001) (Table 2). The process of transformation of chronic to blastic phase become evident by the variation in the nuclear volume. These findings emphasize the capacity of “morphological” recognition by 3D analysis of telomeres, over a population of blast cells, during CML. Verifying the a/c ratio and telomere distribution per nuclear volume, we noted significant difference between the subgroup of samples (Table 2), which might reflect proliferative advantage of blast cells when compared with cells in chronic phase.

**Fig. 4:**
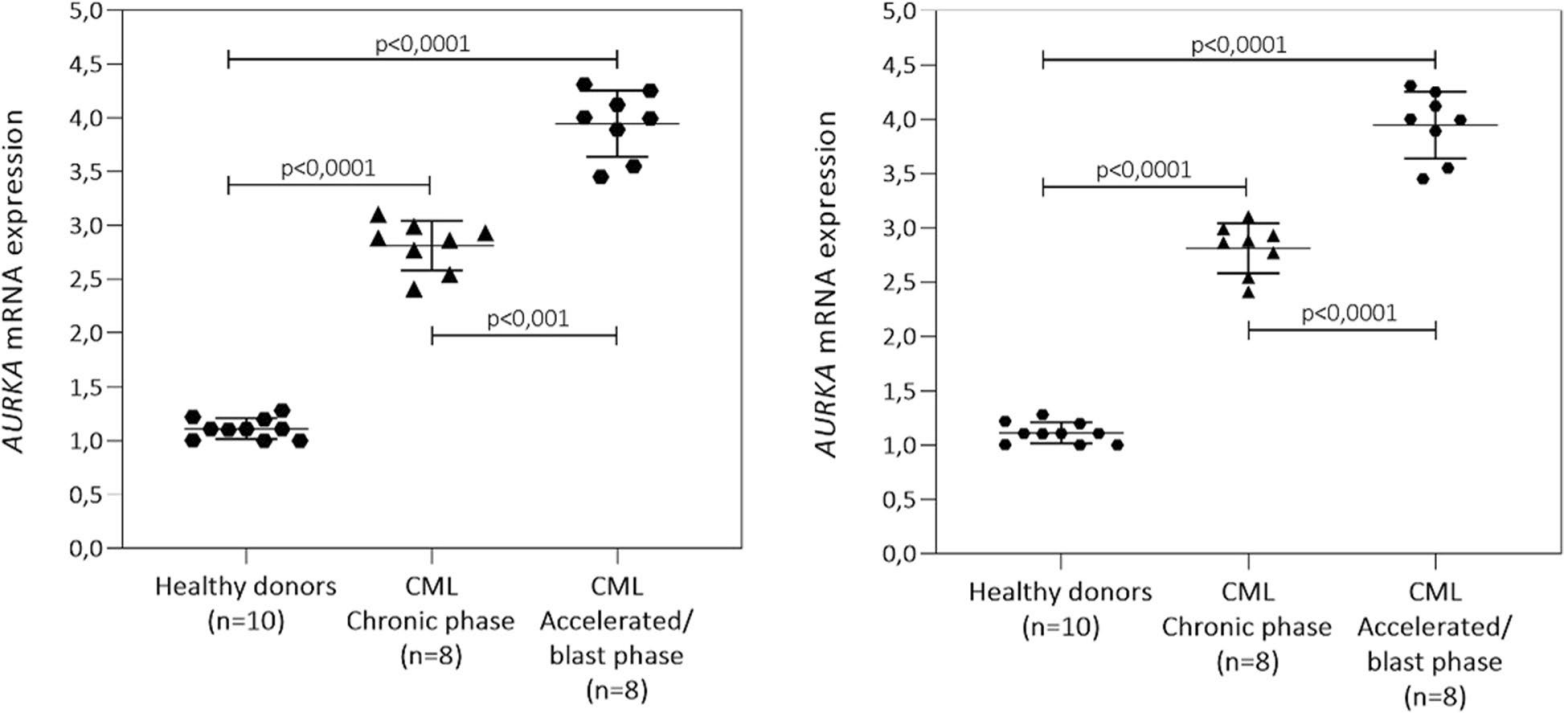
qPCR analysis of *AURKA* and *AURKB* mRNA expression in CML cells. Patients were divided in three distinct sub-groups (*health donors, chronic phase and accelerated phase/blast phase*). The graphs represent the mean ± SD of three independent experiments. The *p* values are indicated in the graphs; *p < 0.05, **p < 0.01; ANOVA test and Bonferroni post-test

We also compared the expression profile of *AURKA* and *AURKB* genes in the two CML subgroups, comparing with healthy donors. However, this strategy was possible to apply in only sixteen samples (CML 01 to CML 08, Table 1). Significant differences were observed (*AURKA* [mean value of 2-∆∆Ct ± SD]: 1.08 ± 0.10 versus 2.85 ± 0.20, *p* < 0.001, in healthy donors versus CML chronic phase cells; *AURKA* [mean value of 2-∆∆Ct ± SD]: 1.12 ± 0.12 versus 4.12 ± 0.22, *p* < 0.001, in healthy donors versus CML chronic accelerated/blast phase (Fig. 4A). The higher expression of *AURKA* in both groups was confirmed by FISH using a commercial probe for *AURKA* gene (*Data not shown*). Using the same stratification criteria for the patients as previously showed (*AURKA*), we found significant differences for *AURKB* (*AURKB* [mean value of 2-∆∆Ct ± SD]: 1.08 ± 0.02 versus 2.62 ± 0.12, *p* < 0.001 in healthy donors versus CML chronic phase cells; *AURKB* [mean value of 2-∆∆Ct ± SD]: 1.06 ± 0.14 versus 3.02 ± 0.21, *p* < 0.001, in healthy donors versus CML chronic accelerated/blast phase (Fig. 4B). The results obtained were confirmed by FISH, by using a commercial probe for *AURKB* gene (*Data not shown*).

## Discussion

Abnormalities related to telomere organization has been described in many cases of cancer [8, 10, 28, 32, 33]. On the other, hand few investigations have explored the telomere organization in CML, regarding its dynamics during the disease progression. For the first time, we compare the telomere organization in two distinct phases of the disease, whose samples became from the same patient. By using 3D nuclear telomeric analysis, it was possible to determine the telomere numbers, the presence of telomere aggregates, telomere signal intensities, nuclear volumes, and nuclear telomere distribution in CML cells. In addition, at the gene expression level, we demonstrated differences in both CML groups, by comparing the expressions of *AURKA* and *AURKB* genes. These parameters, independently, became possible subdivide the CML cells into distinct subgroups. Thus, our results are compatible with the “thesis” that telomere abnormalities generate genomic instability, leading to the CML cells transformation, during the evolution of the disease.

Samassekou et al. (2013) [33] identified abnormal telomere nuclear organization profile on twelve patients diagnosed with CML in chronic phase. According to their analysis the samples were characterized by a high number of telomeric aggregates, and changes in telomeric position. Despite of the absence of correlation with clinical data, Samassekou et al. (2013) [33] confirmed that the telomeres abnormalities observed in CML samples manifested in an early stage of malignancy. Some studies have pointed that telomere from CML cells are shorter than those from healthy leukocytes and are also associated with poor prognostic [34–36]. A high number of telomeric aggregates is closely associated to genomic instability [27].

According to our results the number of telomeric aggregates increases when the disease progress from chronic to accelerated/blastic phase. Therefore, some factors have been proposed to be responsible for telomere abnormalities in CML. The rapid proliferation rate of leukemic cells may represent the force underlying telomere abnormalities. From a not yet clear way, during the disease evolution, telomere abnormalities might be able to induce cell proliferation in one cell lineage, and apoptosis in another cell population, at the same time. In the first situation, the genomic instability gives the cells a proliferative advantage. For the second ones, the genomic instability leads to cell death. This imbalance could explain the clonal expansion and the selective apoptosis in the bone marrow of CML patients [28]. In addition, the elevated activity of tyrosine kinase can generate reactive oxygen species, which are prone to cause damage on telomeres. The occurrence of these events over the course of the disease corroborates for the increase level of genomic instability, making treatment strategies less effective [37].

Previous studies have pointed out that the 3D nuclear telomere abnormalities act as reliable biomarker to predict disease evolution [10, 11, 28, 32]. Our investigation has demonstrated that is possible to distinguish between “cellular status” of lower/elevated level of genomic instability, considering the presence and frequency of telomere aggregates. We found telomere aggregates in all samples. However, significant difference between was observed between chronic and accelerated/blastic phase (Table 2; *p* < 0.001). This evidence makes the nuclear telomere analysis an indicator of disease progression and become the CML an important model to clarify molecular mechanisms underlying tumorigenesis.

Some studies have pointed out that telomere positions, in the nuclei, may act as an important factor beyond chromosome territory. Thus, disruptions in this dynamic process can produce as consequence a differential gene expression pattern, induce chromosomal abnormalities, and disrupt cell function [27, 38–41]. It is possible that in CML, the differential positioning of telomeres may be a consequence of BCR::ABL1 activity, additional chromosomal abnormalities, as seen in different stages of the disease, and gene expression. *AURKA* and *AURKB* mRNA were expressed at significantly higher levels in both CML subgroups, when compared to healthy donors.

The relative expression of *AURKA* and *AURKB* genes, by adopting the mean value from ΔCt, identified two distinct subgroups of CML patients, based on clinical and cytogenetic evidence. Gene amplification may be one, but not the main mechanism leading to overexpression of aurora genes. It’s possible that telomere disruption, in some way, may be related to aurora kinase overexpression and, therefore, to induce mitotic abnormalities. Few years ago, we have demonstrated that *AURKA* and *AURKB* overexpression were associated with genomic instability in cytogenetically stratified group (Normal vs. Abnormal karyotype) of hematopoietic cells and bone marrow derived mesenchymal stem cells (MSCs) of myelodysplastic syndrome patients [42]. However, the regulation of *AURKA* during DNA damage remains most of the time to be well elucidated [22].

Overexpression and amplification of the Aurora kinase genes, particularly *AURKA*, have been documented for many types of neoplasia, with some data evidencing association with clinical parameters, survival, and cancer risk [43]. In human breast cancer, overexpression of these kinases induced aneuploidy, centrosome amplification and tumorigenic transformation. Altered expression of these genes was also reported to correlate with the invasiveness and chromosomal instability of the disease [44]. In agreement, our results suggest that overexpression of *AURKA* and *AURKB* are associated with genomic instability and markers of poor prognosis during CML evolution.

This study demonstrates that 3D telomere organization and the expression levels of aurora kinase genes can be used to subgroup CML patients. Classifying CML patients based on these characteristics might represent an important strategy to define better therapeutic strategies. Our results also suggest an association between progressive telomeric dysfunction and elevated aurora kinase expression, as important components for the evolution of CML. Like previous studies, we found telomere profiles in CML to correlate with distinct clinical phases of the disease [10, 11, 27, 28, 32]. Thus, we propose that 3D telomere organization may be a novel prognostic marker in hematological disorders with define stages driven by genomic instability. However, our study has one limitation, the cohort of patients was relatively small, and we did not determine sorted myeloid cells from bone marrow for all telomere investigations, although previous studies showed that no significant differences in CML telomere lengths are observed when comparing peripheral mononuclear blood cells, fractionated peripheral neutrophils, and non-fractionated bone marrow mononuclear cells [45, 46].

## Data Availability

The datasets used and/or analyzed during the current study are available from the corresponding author on reasonable request.

## Abbreviations

3D: Three-dimensional
CML: Chronic Myeloid Leukemia
TAs: Telomere aggregates
Ph: Philadelphia chromosome
FISH: Fluorescence in situ hybridization
BSA: *Bovine Serum Albumin solution*
SSC: Saline-sodium citrate
Q-FISH: Quantitative fluorescence in situ hybridization
PBS: Phosphate-buffered saline
HCL: Hydrochloric acid
pH: Hydrogen potential
PNA: Pan telomeric probe
cDNA: complementary Deoxyribonucleic acid
RNA: Ribonucleic acid
Ct: Cycle threshold
RQ-PCR: Real-time quantitative polymerase chain reaction

## References

[1] Blasco MA (2005). Telomeres and human disease: ageing, cancer and beyond. Nat Rev Genet.

[2] de Lange T (2005). Shelterin: the protein complex that shapes and safeguards human telomeres. Genes Dev.

[3] de Lange T (2002). Protection of mammalian telomeres. Oncogene.

[4] d'Adda di Fagagna F, Reaper PM, Clay-Farrace L, Fiegler H, Carr P, Von Zglinicki T, Saretzki G, Carter NP, Jackson SP (2003). A DNA damage checkpoint response in telomere-initiated senescence. Nature.

[5] Zhao Y, Sfeir AJ, Zou Y, Buseman CM, Chow TT, Shay JW, Wright WE (2009). Telomere extension occurs at most chromosome ends and is uncoupled from fill-in in human cancer cells. Cell.

[6] Chin L, Artandi SE, Shen Q, Tam A, Lee SL, Gottlieb GJ, Greider CW, DePinho RA (1999). P53 deficiency rescues the adverse effects of telomere loss and cooperates with telomere dysfunction to accelerate carcinogenesis. Cell.

[7] Deng Y, Chan SS, Chang S (2008). Telomere dysfunction and tumour suppression: the senescence connection. Nat Rev Cancer.

[8] Mai S (2010). Initiation of telomere-mediated chromosomal rearrangements in cancer. J Cell Biochem.

[9] Klonisch T, Wark L, Hombach-Klonisch S, Mai S (2010). Nuclear imaging in three dimensions: a unique tool in cancer research. Ann Anat.

[10] Knecht H, Brüderlein S, Mai S, Möller P, Sawan B (2010). 3D structural and functional characterization of the transition from Hodgkin to reed-Sternberg cells. Ann Anat.

[11] Knecht H, Sawan B, Lichtensztejn Z, Lichtensztejn D, Mai S (2010). 3D telomere FISH defines LMP1-expressing reed-Sternberg cells as end-stage cells with telomere-poor 'ghost' nuclei and very short telomeres. Lab Investig.

[12] Vermolen BJ, Garini Y, Mai S (2005). Characterizing the three-dimensional organization of telomeres [published correction appears in cytometry a. 2007 may;71(5):345]. Cytometry A.

[13] Mai S, Garini Y (2006). The significance of telomeric aggregates in the interphase nuclei of tumor cells. J Cell Biochem.

[14] de Klein A, van Kessel AG, Grosveld G, Bartram CR, Hagemeijer A, Bootsma D, Spurr NK, Heisterkamp N, Groffen J, Stephenson JR (1982). A cellular oncogene is translocated to the Philadelphia chromosome in chronic myelocytic leukaemia. Nature.

[15] Lichtman MA (2008). Is there an entity of chemically induced BCR-ABL-positive chronic myelogenous leukemia?. Oncologist.

[16] Perrotti D, Jamieson C, Goldman J, Skorski T (2010). Chronic myeloid leukemia: mechanisms of blastic transformation. J Clin Invest.

[17] Druker BJ (2008). Translation of the Philadelphia chromosome into therapy for CML. Blood.

[18] Quintas-Cardama A, Cortes J (2009). Molecular biology of BCR::ABL1-positive chronic myeloid leukemia. Blood.

[19] Oehler VG, Yeung KY, Choi YE, Bumgarner RE, Raftery AE, Radich JP (2009). The derivation of diagnostic markers of chronic myeloid leukemia progression from microarray data. Blood.

[20] Hans F, Skoufias DA, Dimitrov S, Margolis RL (2009). Molecular distinctions between Aurora a and B: a single residue change transforms Aurora a into correctly localized and functional Aurora B. Mol Biol Cell.

[21] Walter AO, Seghezzi W, Korver W, Sheung J, Lees E (2000). The mitotic serine/threonine kinase Aurora2/AIK is regulated by phosphorylation and degradation. Oncogene..

[22] Krystyniak A, Garcia-Echeverria C, Prigent C, Ferrari S (2006). Inhibition of Aurora a in response to DNA damage. Oncogene..

[23] Vader G, Lens SM (2008). The Aurora kinase family in cell division and cancer. Biochim Biophys Acta.

[24] Ye D, Garcia-Manero G, Kantarjian HM, Xiao L, Vadhan-Raj S, Fernandez MH (2009). Analysis of Aurora kinase a expression in CD34(+) blast cells isolated from patients with myelodysplastic syndromes and acute myeloid leukemia. J Hematop.

[25] Tatsuka M, Sato S, Kitajima S, Suto S, Kawai H, Miyauchi M (2005). Overexpression of Aurora-a potentiates HRAS-mediated oncogenic transformation and is implicated in oral carcinogenesis. Oncogene..

[26] Anand S, Penrhyn-Lowe S, Venkitaraman AR (2003). AURORA-A amplification overrides the mitotic spindle assembly checkpoint, inducing resistance to Taxol. Cancer Cell.

[27] Louis SF, Vermolen BJ, Garini Y, Young IT, Guffei A, Lichtensztejn Z (2005). C-Myc induces chromosomal rearrangements through telomere and chromosome remodeling in the interphase nucleus. Proc Natl Acad Sci U S A.

[28] Gadji M, Adebayo Awe J, Rodrigues P (2012). Profiling three-dimensional nuclear telomeric architecture of myelodysplastic syndromes and acute myeloid leukemia defines patient subgroups. Clin Cancer Res.

[29] Vermolen BJ, Garini Y, Mai S, Mougey V, Fest T, Chuang TC (2005). Characterizing the three-dimensional organization of telomeres. Cytometry A.

[30] de Oliveira FM, Rodrigues-Alves AP, Lucena-Araújo AR, de Paula SF, da Silva FB, Falcão RP (2014). Mantle cell lymphoma harboring Burkitt's-like translocations presents differential expression of aurora kinase genes compared with others 8q abnormalities. Med Oncol.

[31] Sviezhentseva IO, Perekhrestenko TP, Bilko DI, Gordienko AI, Diachenko MV (2015). Dyagil IS3. Functional activity of CD34-positive cells in chronic myeloid leukemia patients with different response to imatinib therapy. Exp Oncol.

[32] Gadji M, Fortin D, Tsanaclis AM, Garini Y, Katzir N, Wienburg Y, Yan J, Klewes L, Klonisch T, Drouin R (2010). Three-dimensional nuclear telomere architecture is associated with differential time to progression and overall survival in glioblastoma patients. Neoplasia.

[33] Samassekou O, Hebert J, Mai S, Yan J (2013). Nuclear remodeling of telomeres in chronic myeloid leukemia. Genes Chromosomes Cancer.

[34] Brummendorf TH, Holyoake TL, Rufer N, Barnett MJ, Schulzer M, Eaves CJ, Eaves AC, Lansdorp PM (2000). Prognostic implications of differences in telomere length between normal and malignant cells from patients with chronic myeloid leukemia measured by flow cytometry. Blood.

[35] Brummendorf TH, Ersoz I, Hartmann U, Bartolovic K, Balabanov S, Wahl A, Paschka P, Kreil S, Lahaye T, Berger U (2003). Telomere length in peripheral blood granulocytes reflects response to treatment with imatinib in patients with chronic myeloid leukemia. Blood.

[36] Drummond M, Lennard A, Brummendorf T, Holyoake T (2004). Telomere shortening correlates with prognostic score at diagnosis and proceeds rapidly during progression of chronic myeloid leukemia. Leuk Lymphoma.

[37] Keller G, Brassat U, Braig M, Heim D, Wege H, Brummendorf TH (2009). Telomeres and telomerase in chronic myeloid leukaemia: impact for pathogenesis, disease progression and targeted therapy. Hematol Oncol.

[38] Pandita TK, Hunt CR, Sharma GG, Yang Q (2007). Regulation of telomere movement by telomere chromatin structure. Cell Mol Life Sci.

[39] Ramirez MJ, Surralles J (2008). Laser confocal microscopy analysis of human interphase nuclei by three-dimensional fish reveals dynamic perinucleolar clustering of telomeres. Cytogenet Genome Res.

[40] Solovei I, Kreysing M, Lanctot C, Kosem S, Peichl L, Cremer T, Guck J, Joffe B (2009). Nuclear architecture of rod photoreceptor cells adapts to vision in mammalian evolution. Cell.

[41] De Vos WH, Hoebe RA, Joss GH, Haffmans W, Baatout S, van Oostveldt P, Manders EM (2009). Controlled light exposure microscopy reveals dynamic telomere microterritories throughout the cell cycle. Cytometry A.

[42] Oliveira FM, Lucena-Araujo AR, Favarin Mdo C (2013). Differential expression of AURKA and AURKB genes in bone marrow stromal mesenchymal cells of myelodysplastic syndrome: correlation with G-banding analysis and FISH. Exp Hematol.

[43] Moore AS, Blagg J, Linardopoulos S, Pearson AD (2010). Aurora kinase inhibitors: novel small molecules with promising activity in acute myeloid and Philadelphiapositive leukemias. Leukemia.

[44] Miyoshi Y, Iwao K, Egawa C, Noguchi S (2001). Association of centrosomal kinase STK15/BTAK mRNA expression with chromosomal instability in human breast cancers. Int J Cancer.

[45] Iwama H, Ohyashiki K, Ohyashiki JH (1997). The relationship between telomere length and therapy-associated cytogenetic responses in patients with chronic myeloid leukemia. Cancer..

[46] Pampalona J, Soler D, Genesca A, Tusell L (2010). Telomere dysfunction and chromosome structure modulate the contribution of individual chromosomes in abnormal nuclear morphologies. Mutat Res.

